# Neuroendocrine carcinoma of the adrenal gland: A rare case report and literature review

**DOI:** 10.1016/j.radcr.2024.06.079

**Published:** 2024-07-30

**Authors:** Achmad Romy Syahrial Rozidi, Wahjoe Djatisoesanto, Ridholia

**Affiliations:** a Department of Urology, Dr. Soetomo Academic General Hospital, Faculty of Medicine, Airlangga University, Surabaya, Indonesia; b Department of Pathological Anatomy, Faculty of Medicine, Universitas Airlangga, Soetomo General Academic Hospital, Surabaya, Indonesia

**Keywords:** Neuroendocrine carcinoma, Adrenal gland, Chemotherapy, Case report

## Abstract

Neuroendocrine carcinoma (NEC) presence in the adrenal glands is rare. Neuroendocrine carcinoma manifests across a wide range of clinical presentations, from asymptomatic cases to those characterized by hormone overproduction or the tumor's mass effect. We report a 48-year-old male referred by a urology specialist with a chief complaint of right-sided back pain for the past 6 months accompanied by nausea, vomiting, and sharp stabbing headaches. The patient had a history of right adrenalectomy surgery. Elevated blood pressure of 150/110 mmHg, and no abnormalities found. The radiologist found a solid lesion and cyst at the lower pole of the right kidney and observed multiple recurrent tumors in the right adrenal on the MRI examination. The biopsy revealed poorly differentiated carcinoma and adrenocortical carcinoma tissue on the second biopsy 2 months later. The patient was diagnosed with neuroendocrine carcinoma; the patient underwent a biopsy guided by CT, followed by a pathological assessment (PA). The surgeon carried out the tumor removal surgery and performed an immunohistochemical (IHC) analysis. A 3-month follow-up is planned to evaluate the potential need for adjuvant chemotherapy. The case underscores the importance of accurate pathological diagnosis and multimodal management in recurrent adrenal tumors, particularly when considering NEC as a differential diagnosis.

## Introduction

Neuroendocrine carcinoma (NEC) originates from neuroendocrine cells and spreads throughout the body. Although tumors can appear in almost any organ, their presence in the adrenal glands is rare. Neuroendocrine carcinoma presents a broad clinical spectrum, ranging from asymptomatic cases to symptoms caused by excessive hormone production or the tumor's mass effect. The pathophysiology of NEC is complex and multifaceted [1]. The molecular mechanisms underlying NEC's development are not fully understood, but various genetic and epigenetic changes have been identified [1], [2], [3], [4].

Management recommendations suggest conducting radiological examinations like CT scans, MRIs of the pelvis or abdomen, and FDG-PET CT scans. Help differentiate whether NEC can be resected, is unresectable, or represents metastasis [5], [6], [7]. Treatment options include chemotherapy, tumor resection, and regular evaluations are essential, with CT scans every 12 weeks for the first year and every 6 months afterwards [8]. This case report presents a rare occurrence of neuroendocrine carcinoma of the adrenal gland treated at a tertiary referral center hospital in Surabaya, Indonesia.

## Cases description

A 48-year-old male was referred by a urologist from the regional government hospital with a diagnosis of right adrenal tumor recurrence. The patient presented to a urology clinic with complaints of intermittent right-sided back pain for the past six months, which worsens when fatigued, along with nausea, vomiting, and sharp stabbing headaches. He had a history of right adrenalectomy surgery in November 2011. However, he did not have the pathological anatomy examination results from the previous hospital. He did not smoke but had hypertension and was taking Candesartan 16 mg and Herbeser 200 mg.

General examination revealed elevated blood pressure of 150/100 mmHg while other vital signs were within normal limits. A physical examination showed a postoperative scar in the right subcostal region region, but no other abnormalities were found. Laboratory investigations yielded normal results (BUN of 14 mg/dL, serum creatinine of 1.1 mg/dL). The cortisol hormone test was normal at 3.8 ug/dL.

**Fig. 1:**
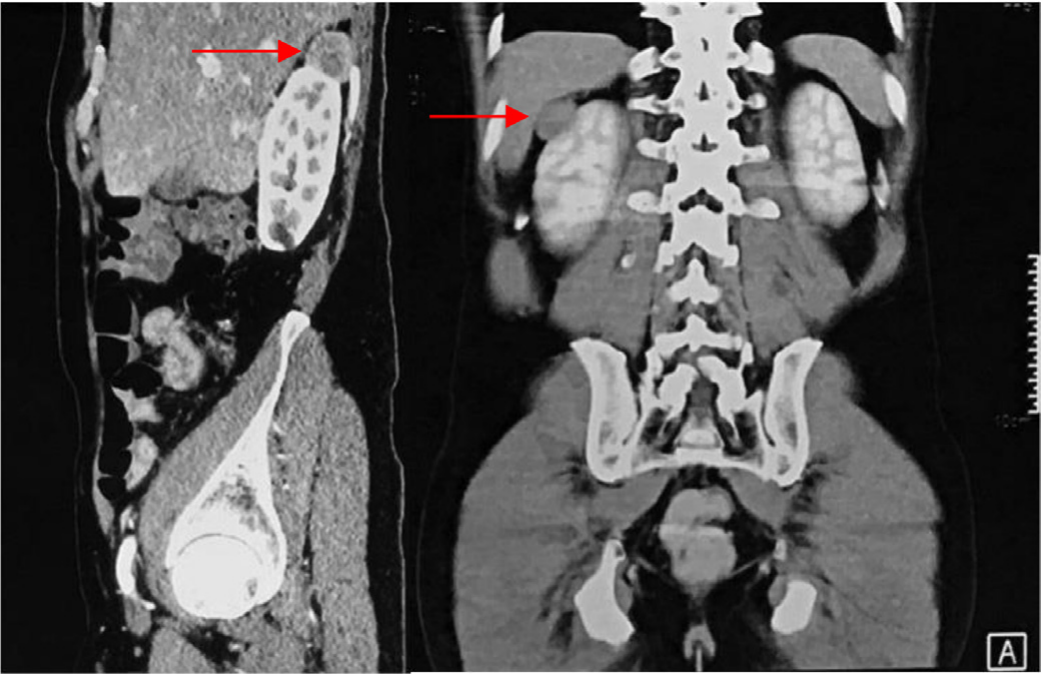
Display of the results of the CT abdomen examination.

**Fig. 2:**
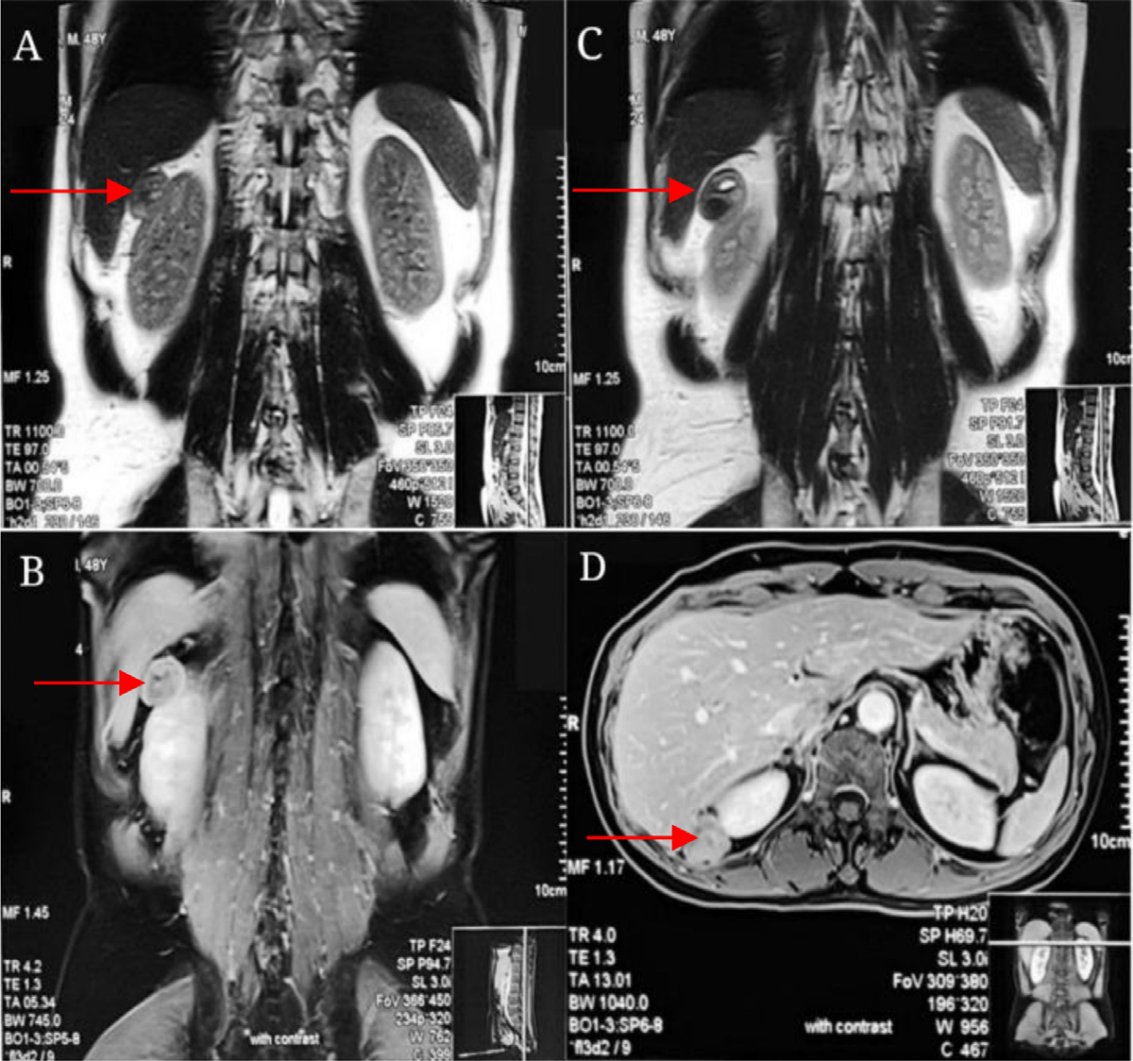
Display of the results of the MRI Abdomen examination with contrast from Dr. Soetomo Academic General Hospital: multiple solid masses measuring 2.9 × 2.7 × 2.9 cm, 1.8 × 2.7 × 2.9 cm, and 4.3 × 1.7 × 4.0 cm in the right adrenal gland.

A single chest X-ray (AP/lateral) showed no signs of metastasis before the operation. The abdominal CT scan with contrast detected a solid lesion measuring 2.77 × 2.65 × 2.3 cm in the right suprarenal area and another lesion measuring 2.41 × 2.74 × 2.12 cm in the anterior side of the right middle pole. Additionally, another cyst measuring 1.7 cm was seen in the lower right renal pole (Fig. 1). Advanced imaging with MRI showed multiple solid masses, the largest being approximately 2.9 × 2.7 × 2.9 cm, 1.8 × 3.5 × 2.4 cm, and 4.3 × 1.7 × 4.0 cm, indicating multiple recurrent tumors in the right adrenal gland (Fig. 2).

**Fig. 3:**
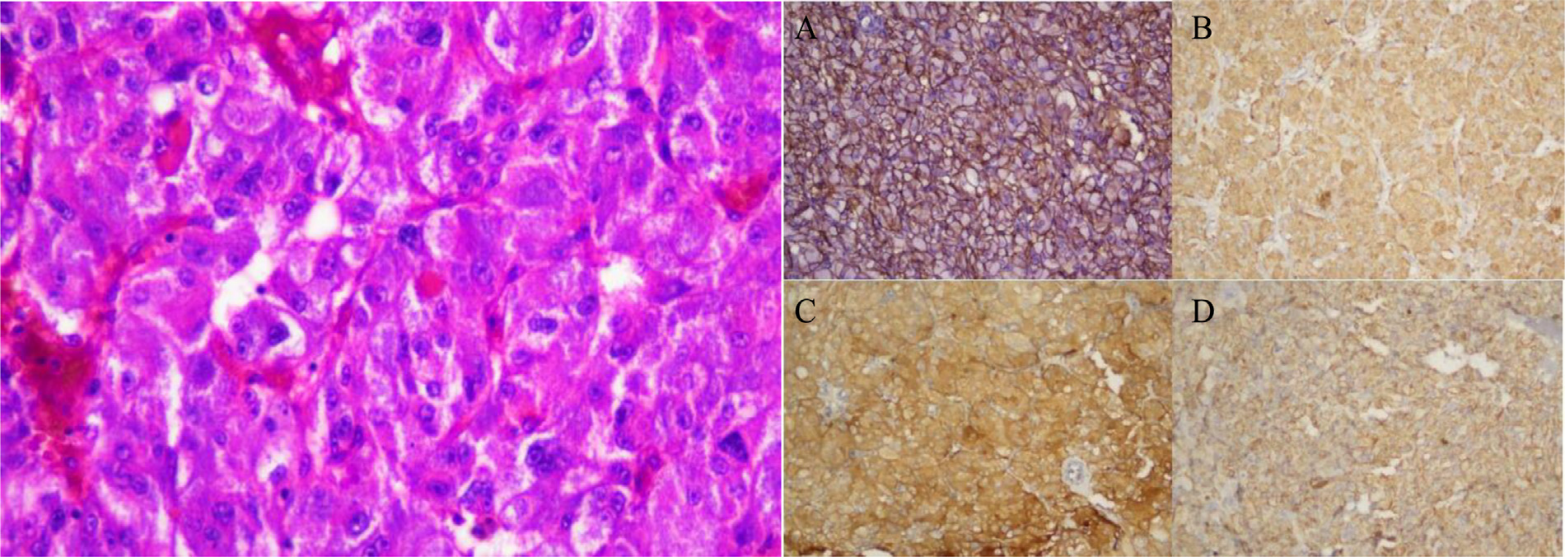
Immunohistochemical examination of the tumor revealed (A) Positive for CD56, (B) Positive for chromogranin, (C) Positive for synaptophysin, (D) Positive for vimentin.

Histopathological examinations revealed poorly-differentiated carcinoma in the recurrent suprarenal area biopsy; b) microscopic findings suggestive of adrenocortical carcinoma from a post-operation tumor tissue; c) immunohistochemical test show negative results for inhibin, calretinin, and CK, which are markers commonly used in the diagnosis of adrenocortical carcinomas, but positive for vimentin, synaptophysin, chromogranin, and CD56 confirming neuroendocrine carcinoma diagnosis (Fig. 3).

**Fig. 4:**
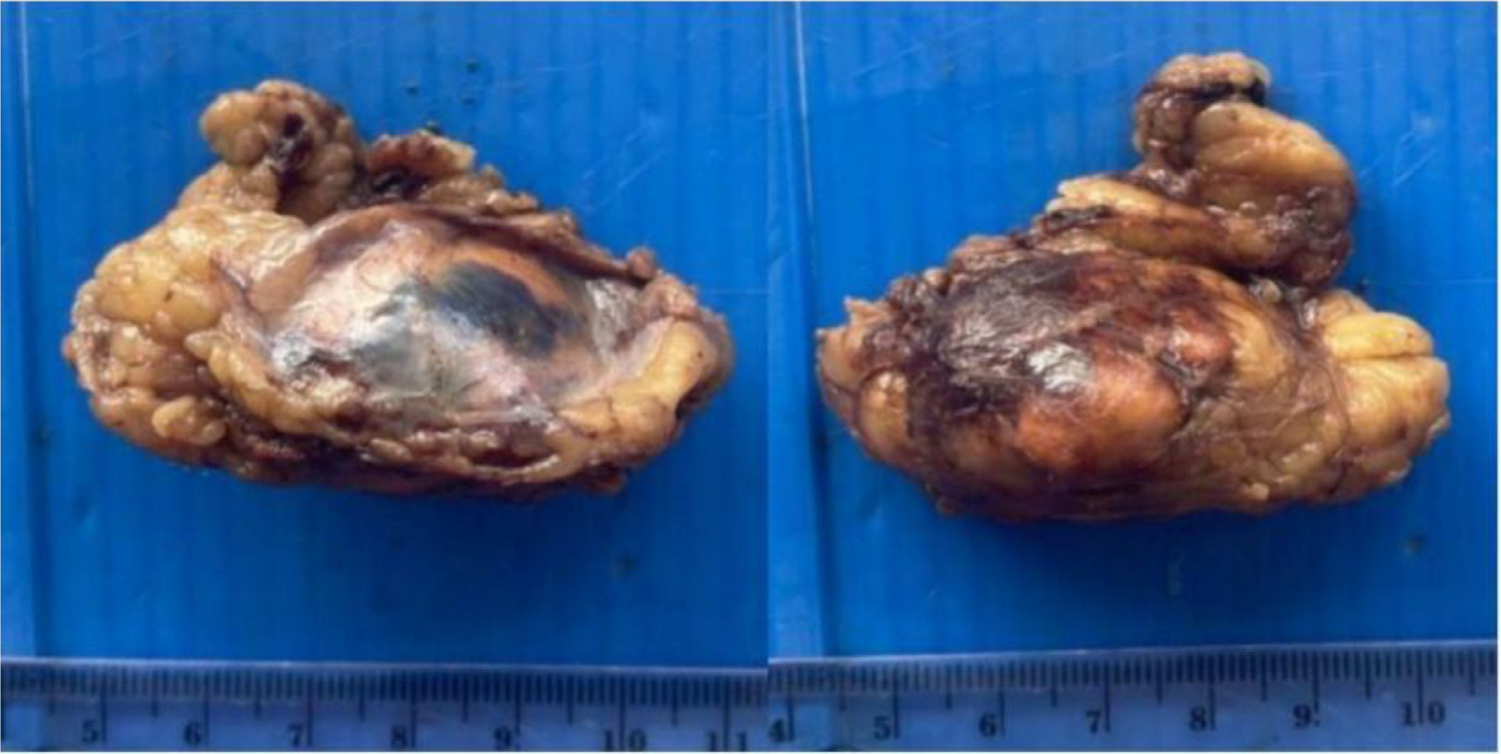
Macroscopic appearance of the tumor after excision surgery.

The patient underwent a CT-guided biopsy, and the pathological examination (PA) revealed characteristics of poorly differentiated carcinoma. Subsequently, an excision surgery was performed on the tumor (Fig. 4), and the PA results indicated adrenocortical carcinoma. Based on these findings, there were suspicions of NEC (neuroendocrine carcinoma). An Immunohistochemical (IHC) examination was conducted to differentiate between adrenocortical carcinoma and NEC. The IHC results indicated that the type of tumor in the patient was indeed NEC. The patient is scheduled for a follow-up 3 months later to evaluate the prognosis and tumor progression using contrast-enhanced abdominal CT scans. At 3-month follow-up, the patient had no complaints and started receiving adjuvant therapy.

## Discussion

Neuroendocrine tumors are most commonly found in the digestive tract, followed by the respiratory system. The appearance of neuroendocrine tumors in other locations is infrequent. Although neuroendocrine tumors can be found in the retroperitoneum, they usually result from metastasis. A primary neuroendocrine tumor in the retroperitoneum itself is infrequent. In the adrenal glands, primary malignant tumors that are often found include adrenal cortex carcinoma, malignant pheochromocytoma, malignant lymphoma, and neuroblastoma [9,2]. Neuroendocrine tumors (NETs) represent a heterogeneous group of tumors that arise from the endocrine cell system distributed throughout the body, especially from the gastro-entero-pancreatic organs. Neuroendocrine tumors are classified based on morphological indices and cell proliferation observed through the Ki67 marker. Well-differentiated NETs are categorized into Group 1 if Ki67 ≤ 2, Group 2 if Ki67 is between 3% and 20%, and Group 3 if Ki67 > 20%. Poorly differentiated NETs are classified as NEC. Patients with NEC often also have pheochromocytoma. Although most pheochromocytomas are benign, there is a possibility of malignancy. However, NEC originating directly from the adrenal gland is an infrequent condition and not fully understood [2].

This case study reports a patient diagnosed with recurrent neuroendocrine carcinoma in the adrenal gland. The patient had a previous history of right adrenal tumors and had undergone an adrenalectomy. In the exact location, based on radiological and histopathological examinations, a recurrent malignancy was found, leading to the excision of the recurrent tumor in the patient. Local resection involving surrounding structures, including vascular and lymph nodes, was performed [8]. Most extrapulmonary NECs are aggressive, requiring multimodal management. Moreover, NEC in the adrenal gland is rarely found and often does not involve symptoms of hormonal disorders, indicating a nonspecific clinical picture. Upon radiological evaluation, no metastasis was found in this patient; hence, no adjuvant therapy was administered post-resection.

According to consensus [10] for patient with clinically/biochemically suspected NEC (not pathologically verified), MRI abdomen or contrast-enhanced triple phase CT and PET/CT with [^68^Ga]Ga-DOTA-SSA (including [^68^Ga]Ga-DOTA-TOC, [^68^Ga]Ga-DOTATATE, and [^68^Ga]Ga-DOTA-NOC) were chosen. [^68^Ga]Ga-DOTA-SSA PET/CT was preferred for SSTR imaging to stage all NEC, complementing conventional imaging. SSTR imaging, CT, and/or MRI were required for all patients at initial or subsequent staging. [^68^Ga]Ga-DOTA-SSA PET/CT was recommended for NEC patients with metastatic disease but uncertain primary tumour location. SSTR imaging was considered necessary at re-staging after potentially curative surgery in patients with clinically significant risk of residual or metastatic disease as a supplement to conventional imaging, even if no SSTR imaging was done before surgery to confirm SSTR expression. All patients required SSTR imaging for re-staging after noncurative surgery as a supplement to conventional imaging. In this case, [^68^Ga]Ga-DOTA-SSA not performed due to resources limitation. PET imaging is quite valuable in differentiating between benign NETs and NECs. Although not all centres have access to PET, there are situations where clinicians must differentiate lesions only using an alternative imaging modality. Generally, neuroendocrine tumours (NETs) are characterised by a strong blood supply and a distinct boundary, often causing displacement of nearby tissues without entering them. This is usually determined by the radiographic features. In contrast, carcinomas often exhibit a more aggressive and uneven morphology. Computed tomography (CT) and magnetic resonance imaging (MRI) are 2 imaging techniques that can be useful for distinguishing between the 2 in this specific scenario. NETs frequently display uniform enhancement on CT scans and seem hypointense on T1-weighted MRI images. However, carcinomas might exhibit varied amplification and increased brightness on T1-weighted MRI scans. Although ultrasonography (USG) can be used to detect neuroendocrine tumours (NETs), particularly in the liver, it is less effective in differentiating them from carcinomas [11]. In a study by Ogawa et al. [12], a 79-year-old male patient with a history of diabetes mellitus presented with abdominal pain. An abdominal CT scan revealed a 51 × 36 mm heterogeneous mass in the left adrenal gland with normal hormonal tests. Two months later, an evaluation CT scan showed the mass increased to 68 × 52 mm without tumor metastasis. A laparoscopic adrenalectomy was performed, revealing a whitish tumor tissue with necrosis. Immunohistochemical examination showed tumor cells positive for CD56 and synaptophysin, indicating small-cell neuroendocrine carcinoma. Postoperative CT scan evaluation revealed metastases in lymphatic tissues, the right adrenal gland, the liver, and lymph nodes at the left kidney hilum. Eleven months post-operation, the patient's condition deteriorated, leading to death due to cancer, presented in Table 1 [12].

**Table 1:** Summary of case report journal regarding neuroendocrine carcinoma.

Author	Cases	CT-Scan	IHC Examination	Management
Ogawa et al. [12]	a 79-year-old male patient with a history of diabetes mellitus presented with abdominal pain	Heterogeneous mass measuring 51 × 36 mm in the left adrenal gland.	Small-cell NEC with tumor cells positive for CD56 and synaptophysin.	Laparoscopic adrenalectomy.
Limonnik et al. [13]	a 62-year-old male with a history of hypertension presented with back pain, a 25 kg weight loss, decreased appetite, and night sweats for a month.	Retroperitoneal mass measuring 18.3×12.2 cm in the left adrenal gland.	NEC with tumor cells positive.	Tumor resection in the spleen, left kidney, adrenal gland, and distal pancreas with neoadjuvant chemotherapy.

In another case report, a 62-year-old male with a history of hypertension presented with back pain, a 25 kg weight loss, decreased appetite, and night sweats for a month. The abdominal CT scan showed a retroperitoneal mass measuring 18.3 × 12.2 cm in the left adrenal gland. Biopsy results indicated NEC with tumor cells positive on immunohistochemical examination. The patient underwent neoadjuvant chemotherapy, resulting in a significant reduction in mass size. Subsequently, the patient underwent tumor resection in the spleen, left kidney, adrenal gland, and distal pancreas. Upon evaluation, no recurrence was observed, presented in Table 1 [13].

In another study, Chang et al. [14] conducted a retrospective review of patients diagnosed with small cell carcinoma in the genitourinary organ, finding only 1 patient with a primary tumor from the adrenal gland. However, the tumor in this patient had metastasized. Currently, there are no standard treatment guidelines for neuroendocrine carcinoma in the adrenal gland, partly due to the rarity of the disease. NCCN provides guidelines for resectable extrapulmonary operations, but for poorly differentiated neuroendocrine carcinoma, the therapeutic options are numerous and depend on the disease's location. Treatment options include different combinations such as tumor resection, neoadjuvant or adjuvant chemotherapy with cisplatin/etoposide, carboplatin/etoposide, FOLFOX, FOLFIRI, and radiation therapy [8].

## Conclusions

Neuroendocrine carcinoma in the adrenal gland poses a rare and challenging clinical scenario, often lacking specific symptoms and showing aggressive behavior. The cases discussed underscore the importance of timely diagnosis and multimodal management approaches due to the high propensity for recurrence and metastasis. Surgical resection remains key, but standardized treatment guidelines are lacking due to its rarity. Multimodal management with adjuvant therapies such as chemotherapy and radiation therapy should be considered, emphasizing the need for individualized approaches. Further research and collaborative efforts are warranted to elucidate optimal therapeutic strategies and improve outcomes for patients with neuroendocrine carcinoma originating from the adrenal gland.

## Patient consent

Informed consent for patient information to be published in this article was obtained. Appropriate informed consent was obtained for the publication of this case report and accompanying images.

## Ethical approval

This report has been approved by the ethical committee of Dr. Soetomo General-Academic Hospital.
